# Methylphenidate can help reduce weight, appetite, and food intake—a narrative review of adults’ anthropometric changes and feeding behaviors

**DOI:** 10.3389/fnut.2024.1497772

**Published:** 2024-11-29

**Authors:** Fernand Vedrenne-Gutiérrez, Sion Yu, Anna Olivé-Madrigal, Vanessa Fuchs-Tarlovsky

**Affiliations:** ^1^School of Medicine and Health Sciences, Universidad Anáhuac, Mexico City, Mexico; ^2^Department of Clinical Nutrition, Hospital General de México Eduardo Liceaga, Mexico City, Mexico

**Keywords:** methylphenidate, obesity, feeding behaviors, appetite, weight

## Abstract

**Introduction:**

Obesity constitutes a complex global health that carries several comorbidities that include cardiovascular disease, diabetes, and cancer. Current treatments, such as lifestyle modifications and bariatric surgery, are often difficult to implement or carry risks, creating a need for alternative approaches. Methylphenidate (MPH), a drug commonly used to treat Attention Deficit and Hyperactivity Disorder (ADHD), has shown potential in regulating dopamine levels to modulate appetite and feeding behaviors.

**Methods:**

This narrative review evaluated the effect of MPH in reducing food intake, body weight, and anthropometric indicators in adults with obesity or overweight. Using the PICO method, 39 studies were selected, including 14 randomized controlled trials and 3 observational studies.

**Results:**

MPH canblead to modest weight loss of 1–2% and significant appetite suppression, with stronger effects observed in women, who reported greater reductions in appetite and food cravings. Studies could remain underpowered to detect consistent effects in men.

**Discussion:**

Even if these results suggest MPH could be an option for treating obesity, concerns regarding its safety profile and long-term efficacy persist. This review underscores the need for further investigation to confirm MPH’s therapeutic potential, particularly through studies that address gender-specific responses and evaluate its sustainability as a weight management tool.

## Introduction

Obesity has become a major pandemic of the 21st century (1, 2). Being overweight leads to being in a chronic state of inflammation, which increases the risk of many serious health problems, including heart disease, stroke, diabetes, and cancer (3, 4). Obesity also takes an economic toll, with billions spent each year on obesity-related medical costs (1). Despite this, obesity can be categorized as one of the most refractory conditions since lifestyle changes like diets and exercise are challenging to maintain long-term in the actual fast-paced world (5–7). Irreversible treatments such as bariatric surgeries are effective. Still, they carry risks and are only suitable for selected patients (8). There is an urgent need for additional interventions to aid individuals in achieving and maintaining a healthy body weight. Pharmacological treatments targeting the biological mechanisms of obesity could serve as a critical enhancement to the existing therapeutic arsenal.

The Mesolimbic Dopaminergic Pathway, established in the ventral tegmental area (VTA), is a fundamental regulator of the brain’s reward system, coordinating pleasure and reinforcement learning through various other neural pathways (9). Its primary neurotransmitter, dopamine, transmits signals associated with reward-related stimuli from the VTA to crucial brain regions such as the nucleus accumbens (NAc), amygdala, and prefrontal cortex (10). When individuals participate in pleasurable activities, for example, consuming food, dopamine is released in the NAc, triggering the feeling of satisfaction, reinforcing positive feedback for motivation, and a sense of reward. This process enhances motivation and facilitates learning by associating specific actions with positive outcomes, thus shaping future behaviors (11). In individuals with obesity, the mesolimbic dopaminergic system may be dysregulated. Naef et al. explained that these individuals showed reduced dopamine D2 receptor availability in the striatum, suggesting a hypodopaminergic state and resulting in overconsumption of food to compensate for reduced dopamine signaling (12). Drugs that modulate dopamine neurotransmission could help restore normal function in this system, consequently eating less and losing weight (13).

**Figure 1:**
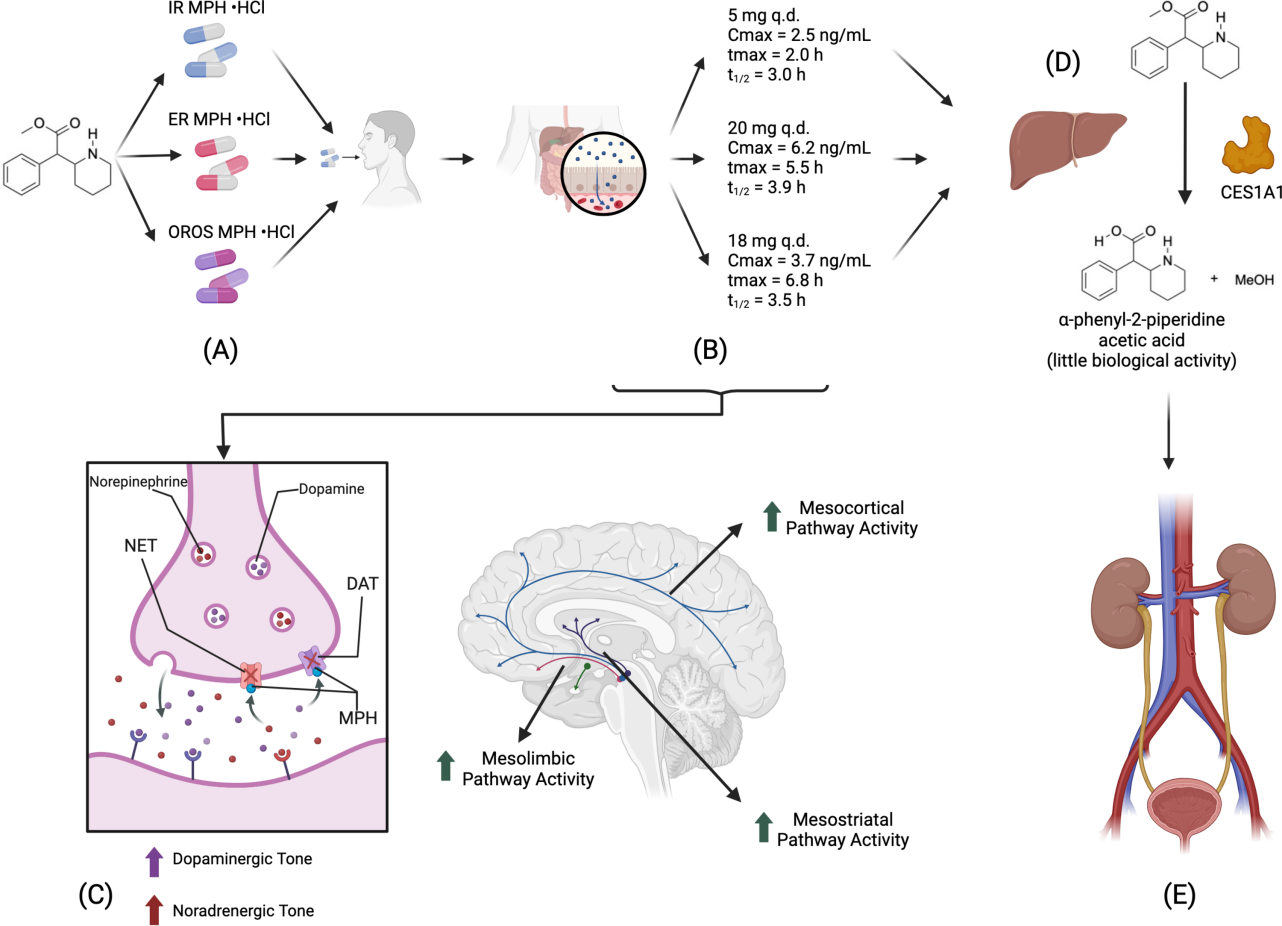
Pharmacokinetics and pharmacodynamics of MPH. **(A)** MPH exists in immediate (IR), extended-release (ER), and osmotic release oral systems (OROS). **(B)** MPH is readily dissolved in water. It is not absorbed in the stomach but absorbed in the intestine. IR requires to be given tid to have pharmacokinetics similar to ER and OROS. **(C)** Once distributed, MPH can cross the blood–brain barrier (BBB). It inhibits Dopamine (DAT) and Norepinephrine (NET) transporters, thus increasing the activity of noradrenergic and dopaminergic pathways. **(D)** MPH is metabolized into *α*-phenyl-2-piperidine acetic acid by Carboxylesterase-1 (CES1A1). This metabolite has deficient biological activity and has renal elimination (12).

Methylphenidate (MPH) is a central nervous system stimulant that increases levels of dopamine and norepinephrine in the brain by inhibiting its reuptake in the presynaptic neuron. In so doing, MPH increases dopaminergic transmission in the mesolimbic (ML), mesocortical (MC), mesostriatal (MS), and infundibular (IN) pathways. Methylphenidate is metabolized in the liver and is readily eliminated through the kidneys (14) (Figure 1). MPH is primarily used to treat attention-deficit hyperactivity disorder (ADHD). Still, it has also been investigated for its potential weight loss effects by increasing dopaminergic activity in the ML, MC, and MS pathways and, ultimately, the reward system (15).

Lifestyle changes should remain the primary line of obesity treatment. However, medications could play a crucial role in aiding appetite control. Drugs that target the dopaminergic reward system could help people lose weight and maintain their long-term health (16). As mentioned before, MPH is one potential candidate; nevertheless, more research must be done to be approved by the FDA (17, 18). Other drugs that modulate dopamine, such as antidepressants and anxiolytics, are also being investigated (16). Ultimately, lifestyle changes, behavioral therapy, and pharmacotherapy may be the most effective approach to the obesity pandemic (19). Medications could be an essential tool to help people lose weight and improve their health (20). With further research and development, we may see more anti-obesity drugs approved in the coming years. The main objective of this narrative review is to examine the current literature on the effects of methylphenidate (MPH) on appetite suppression and weight regulation in adults with obesity or overweight.

## Methods

**Figure 2:**
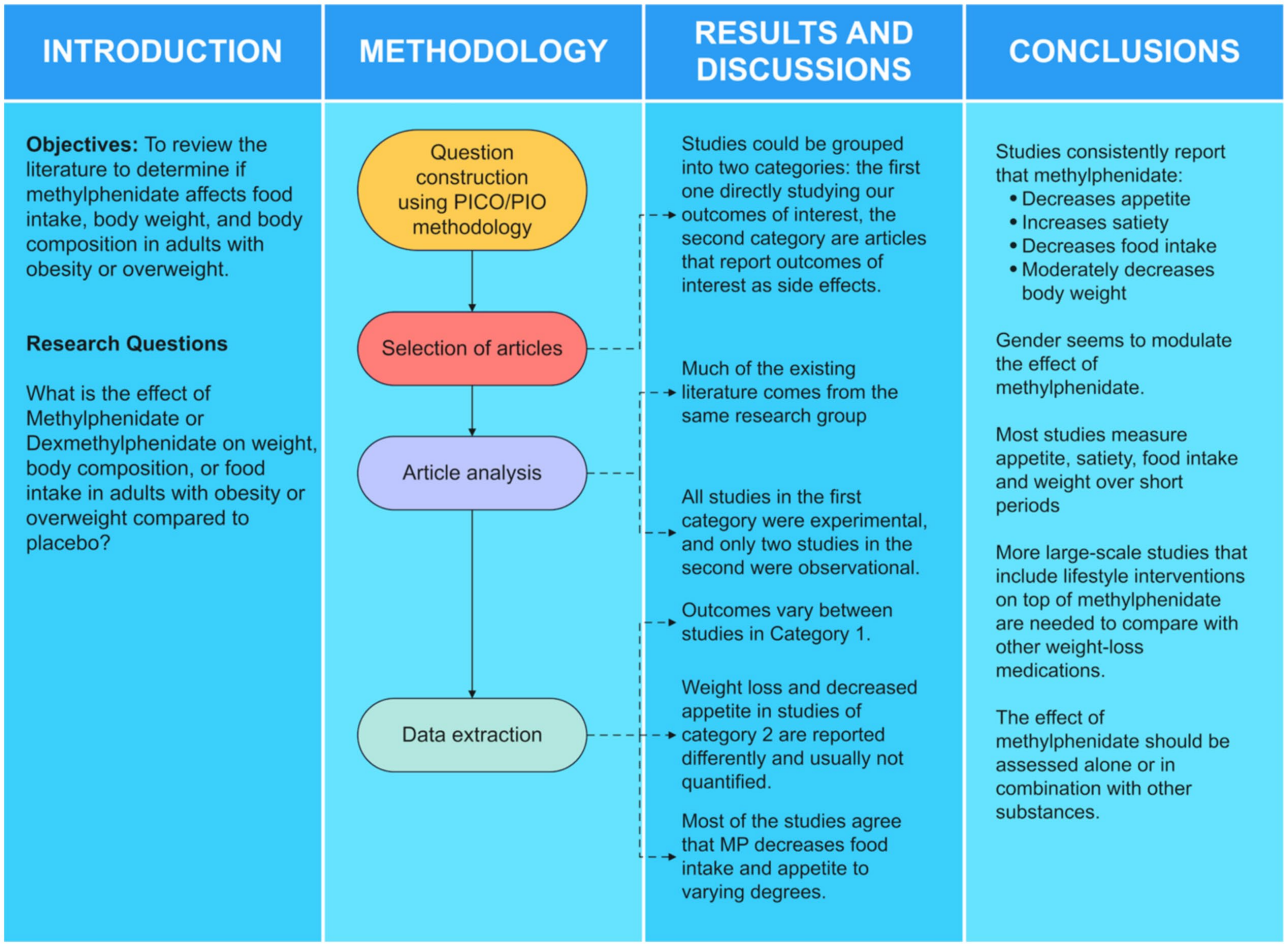
Methodology roadmap—this study followed the steps above to answer our research question. The results can be analyzed at different descriptive levels: the type of articles found, including their design, and the actual data in the literature.

**Figure 3:**
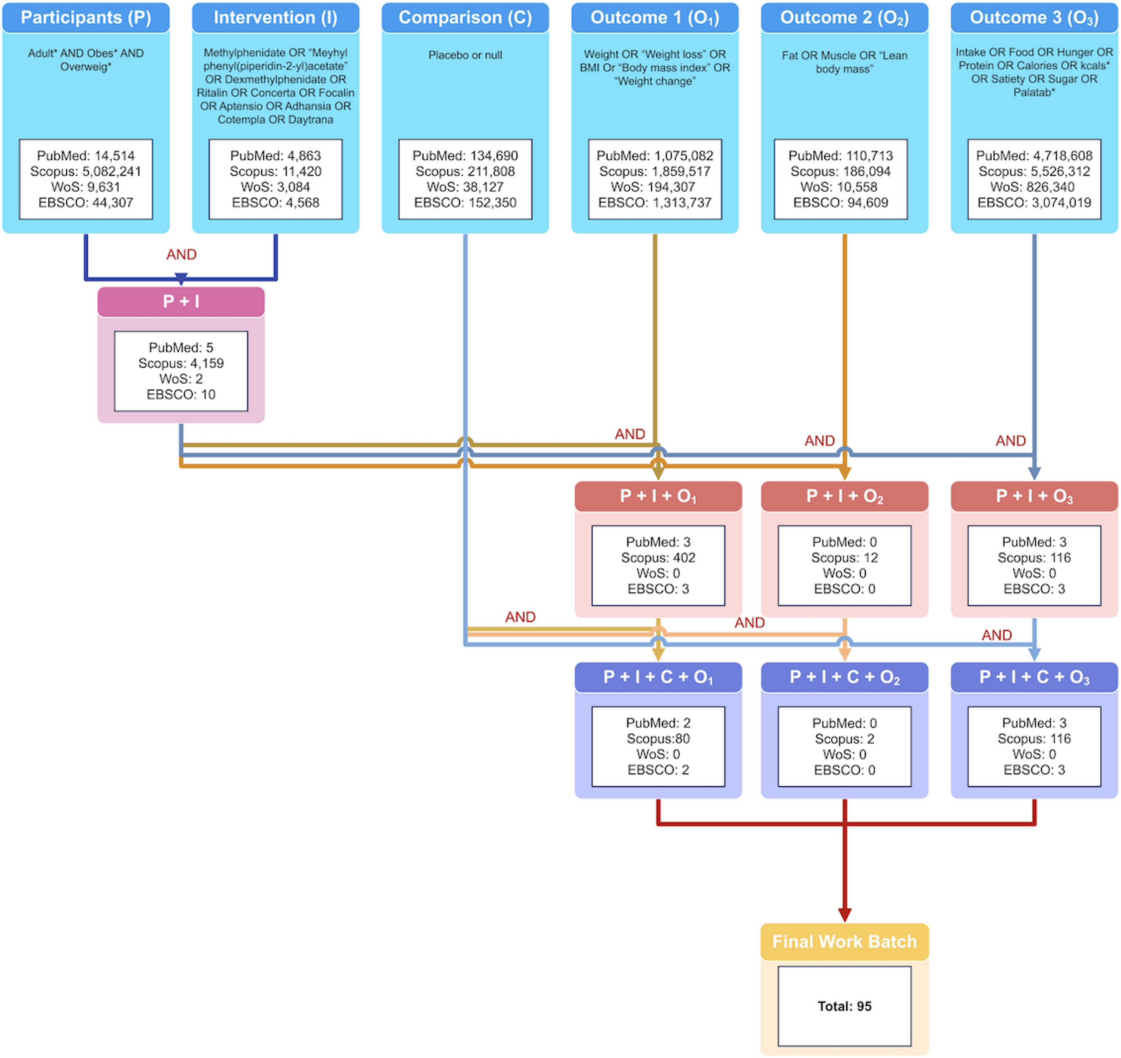
PICO/PIO methodology decision tree—several search queries were built using Boolean operators to reach a final work batch of 95 further screened articles.

To perform this review, a Participant-Intervention-Comparison-Outcome (PICO) approach was followed to answer our research question. A methodological roadmap is shown in Figure 2. We present a decision tree in Figure 3 to show how the search queries were built. Six different search queries (Figure 3) were used in 4 databases: PubMed, Scopus, Web of Science, and EBSCO. These databases were chosen because of the scope and breadth of journals they cover. We included only articles published in English after 2010 to cover all the relevant publications in the last 10 years. Studies had to be experimental and observational studies in human adults that reported objective anthropometric, appetite, or dietetic indicators or that reported weight loss as a side effect of MPH. MPH dosage had to be disclosed. Reviews, meta-analyses, conference papers, animal models, *in-vitro* studies, studies in children, articles published before 2010, articles without relevant outcomes, with patients receiving a mix of medications, or where participants had any condition that could produce weight loss were excluded.

**Figure 4:**
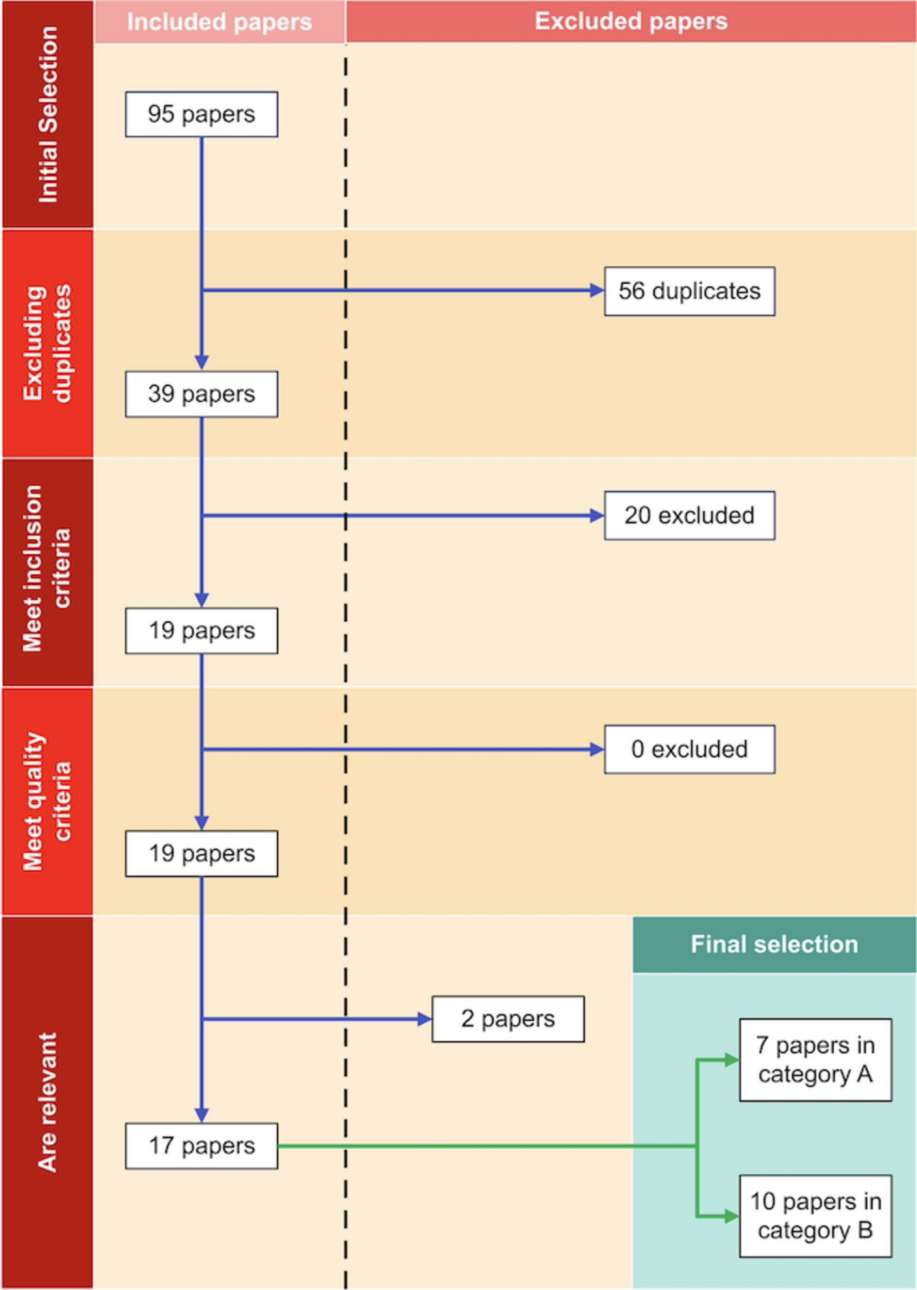
PRISMA flowchart depicting the process of article selection.

A total of 39 articles were selected (Figure 4). Articles could be grouped into two categories: category 1 had articles that addressed our research question directly, and category 2 had articles that reported weight loss, appetite changes, and other side effects related to nutrition status because of MPH when used for other purposes. Out of the 39 articles, 17 met the inclusion and exclusion criteria to different extents. Of the 39 selected articles, 33 (85%) were experimental or observational, 34 (90%) were carried out on human adults, all of them were published after 2010, 26 (67%) had a relevant anthropometric or appetite outcome, 32 (82%) had a methylphenidate dose declared, all of them were in English or Spanish (100%), 3 (8%) used different medications. In none of the articles did participants have other weight loss predisposing conditions. The most common reason for rejecting an article was that articles did not declare anthropometric or appetite outcomes. The studies varied in design and size, but the majority (83.3%) were randomized controlled trials (RCTs). The remaining articles were all cohort studies. Seven studies (41.2%) were grouped in category 1, while the remaining 10 (58.8%) could be grouped in category 2.

## Results

### Effects of MPH on body weight, eating behaviors, and appetite

The present review looked at studies assessing the effects of methylphenidate (MPH) on various anthropometric and behavioral outcomes related to weight management, including body weight, eating behaviors, and appetite in adults. Only half (*n* = 741.2%) of the selected studies belonged to category 1 (18, 21–26). Weight and Body Mass Index (BMI) and waist circumference were the only studied anthropometric outcomes. Weight was an outcome in 4 studies (57.1%) (18, 24, 26), BMI was an outcome in 2 studies (28.6%) (24, 25), and waist circumference was an outcome in only one study (14.3%) (24). Only two studies (28.6%) found that MPH had a significant effect on anthropometric indicators: Heffner et al. (26) found a 1.6% weight decrease in participants who were trying to quit smoking and took MPH versus a 1.3% weight increase in participants who were trying to quit smoking in the placebo group (*p* < 0.001); on the other hand, Quilty et al. (25) showed that when compared to cognitive behavioral therapy (CBT), treatment with MPH produced a more considerable decrease in BMI (*p* = 0.01) (Table 1).

**Table 1:** Summary of articles that looked at dietetic or anthropometric as a function of MPH use.

Article	Country	Design	Objective	Sample characteristics	Intervention	Relevant outcomes	Main findings	Methodological remarks
Davis et al. (21)	Canada	Cross-over Randomized Controlled Trial	To assess the effect of BMI and gender on food consumption, food cravings and appetite after administering Methylphenidate (MPH)	*n* = 132 Adults between 24–45 years old. 73.5% female No history of DSM IV Axis I disorders (except unipolar depression). No history of serious medical illness. Not taking medications contraindicated against methylphenidate. 44% of the sample had BMI < 25. 56% had BMI >30. 19% smoked tobacco.	Patients were given short-acting 0.5 mg/kg of MPH as intervention. After 1 h, they were presented with their favorite snack in two occasions (one placebo, one MPH).	Appetite Rating: validated own instrument with 3 questions. Food Cravings: General Food cravings questionnaire %Snack Food Consumption: In-lab feeding test.	Snack consumption was equivalent among both genders, BMI categories, and their interactions. Normal weight individuals significantly decreased their appetite rating (*p* = 0.017), food cravings (*p* < 0.0001), and snack consumption (*p* < 0.017) regardless of gender. In individuals with obesity, there was a significant gender x day in appetite ratings (*p* < 0.007), food cravings (*p* = 0.008), and snack consumption (*p* < 0.0001). No changes in appetite ratings, food cravings, or snack consumption were seen in males, but they were seen in females (*p* < 0.0001 for all).	While the study has a large sample size. Most participants were females, so the male group may be underpowered to find statistical significance.
Davis et al. (23)	Canada	Cross-over Randomized Controlled Trial	To assess whether food addiction status and gender modulate food consumption, food cravings and appetite after administering MPH	*n* = 136 Adults between 25 and 50 years old. Predominantly overweight or with obesity. 67.7% female 17% met criteria of food addiction according to YFAS. Mean BMI of food addiction group did not differ from that of the rest of the group. No history of serious medical illness, psychotic disorders, or substance abuse disorders. Not taking medications contraindicated against methylphenidate. 26 and 20% of the participants with food addiction and the general group smoked tobacco, respectively.	Patients were given short-acting 0.5 mg/kg of MPH as intervention. After 1 h, they were presented with their favorite snack in two occasions (one placebo, one MPH)	Appetite Rating: validated own instrument with 3 questions. Food Cravings: General Food cravings questionnaire %Snack Food Consumption: In-lab feeding test. Food addiction: YFAS questionnaire.	Participants in the food addiction group had higher baseline food craving scores and appetite ratings (*p* < 0.0001 for both). There was a decrease in appetite ratings and craving scores between placebo day and MPH Day (η^2^ = 0.157, *p* = 0.031) and (η^2^ = 0.128, *p* = 0.006 respectively). The interaction between placebo/MPH and food addiction was not statistically significant. The interaction between placebo/MPH and food addiction was significant for food consumption (*p* = 0.018). The food addiction group did not decrease their food consumption, but the general group did (η^2^ = 0.276 p < 0.0001). Women also tended to consume less of their snack than men (η^2^ = 0.039, *p* = 0.022).	It is possible that the food addiction group was underpowered to produce significant differences in variables, so results must be interpreted with caution, even if the study itself has a large sample size.
Davis et al. (22)	Canada	Cross-over Randomized Controlled Trial	To assess whether having binge eating disorder (BED) modulates food consumption, appetite, and food cravings after administering MPH	*n* = 198 Adults between 24 and 50 years old. All of them with overweight or obesity. 76.8% female 96 participants had binge eating disorder (76 females). No history of serious medical illness, psychotic disorders, or substance abuse disorders. Not taking medications contraindicated against methylphenidate (MPH).	Patients were given short-acting 0.5 mg/kg of MPH as intervention. After 1 h, they were presented with their favorite snack in two occasions (one placebo, one MPH).	Appetite Rating: validated own instrument with 3 questions. Food Cravings: General Food cravings questionnaire %Snack Food Consumption: In-lab feeding test.	Self-reported appetite (*p* = 0.002), food cravings (*p* = 0.023), and snack consumption (*p* = 0.002) decreased significantly between placebo day and MPH Day. There was also a significant day x sex interaction (*p* = 0.007, *p* = 0.048, and *p* = 0.032 respectively), showing only a decrease in female participants (*p* < 0.0001 in all cases). BED status did not modulate the response.	While the study has a large sample size, most participants were females, so lack of significance in the male population should be taken with caution due to possible underpowering.
El Amine et al. (18)	Canada	Randomized Controlled pilot Trial	To determine the effect of short-acting MPH at 0.5 mg/kg during 2 months on appetite sensations, olfactory threshold, energy intake, and body weight in individuals with obesity	*n* = 12, randomized into a placebo group with *n* = 7 (3 males and 4 females), and an MPH group with *n* = 5 (2 males and 3 females). Adults between 18 and 40 years old with BMI > 30 kg/m^2^ but body weight below 200 kg so as not to surpass the maximal dose of MPH (100 mg/d). All had a stable weight for the past 6 months. None of them smoked, had ADHD, used MPH, had history of mental health or substance abuse disorders, took any medication that could affect appetite, had any major health problem, or reported any food allergy	Patients received short-acting 0.5 mg/kg of MPH or placebo divided twice daily 1 h after lunch and dinner. One initial appointment and two measuring appointments were scheduled monthly.	Appetite: Visual Analog scale (desire to eat, hunger, prospective food consumption, and fullness). Olfaction: Sniffin’ sticks^®^. Bodyweight Height Body composition: DXA. Energy intake: In-lab feeding test.	For olfaction, there is a significant interaction in group x time (*p* = 0.029), where participants receiving MPH increased their olfaction threshold (*M* = −3.8, *p* = 0.017). There was a significant decrease in the areas under the curve for desire to eat (*p* = 0.001), hunger (*p* = 0.008), and prospective food consumption (*p* = 0.003); and an increase in fullness (*p* = 0.028) in the MPH group when compared to placebo. Changes in olfaction and appetite variables were not correlated with anthropometric variables.	Sample size is small and thus not generalizable; however, these results look promising for a larger scale study.
Goldfield et al. (24)	Canada	Cross-over Randomized Controlled Pilot Trial	To estimate if there is gender modulates the effect of short-acting 0.5 mg/kg MPH on energy intake, macronutrient consumption, food preferences, appetite sensations and relative reinforcing value of food.	*n* = 120 Adults between 18 and 40 years old with BMI larger or equal than 20 kg/m^2^ but body weight less than 120 kg to not surpass maximal dose of MPH. 50% female All non-smokers and non-tobacco users.	Patients received short-acting 0.5 mg/kg MPH at sessions. One initial appointment, and two subsequent monthly (for females) or weekly (for males) appointments for measurements. Participants had to eat from a standardized mixed meal buffet 1 h after taking the pill.	Appetite variables: Visual analog scale (desire to eat, hunger, prospective food consumption, and fullness) Buffet Energy and micronutrient Intake Weight Height Waist Circumference BMI Red button pressing for relative reinforcing value of food.	Significant gender x drug interaction for energy intake (*F* = 4.9, *p* = 0.01) and carbohydrate intake (*F* = 8.2, *p* = 0.02) with a greater reduction in men than in women relative to placebo. No significant gender x drug interaction for macronutrient preferences. No drug x gender interaction for food hedonic ratings, relative reinforcing value of food, and water intake in the buffet test. No drug x gender interaction for satiety quotients of appetite sensations. Hunger ratings between MPH and placebo groups were not statistically different before or after drug administration.	This trial has a large sample size with equal gender representation.
Heffner, 2013 (26)	USA	Randomized Controlled Trial	To study the effect of Osmotic Release Oral System (OROS)-MPH on weight gain of quitting smokers with ADHD.	*n* = 215 Adults 18–55 years old. Smoking at least 10 cigarettes/day, expired CO level ≥ 8 ppm, DSM-IV ADHD Rating Scale score > 22. In good physical and mental health; no narrow angle glaucoma, tics, seizure disorder, Tourette syndrome. Non-nicotine substance abuse, mood/anxiety disorders, antisocial personality disorder, psychosis. Without recent treatment for smoking or ADHD	OROS-MPH was titrated to a dose of 72 mg/day over the first 2 weeks and continued at the maximum tolerated dose until the end of the 11-week treatment period. Participants had 11 appointments once every week. In each visit, participants received counseling and a nicotine patch. Weight assessments were conducted at baseline, week 6, and week 11.	ADHD diagnosis or severity: Adult ADHD Clinical Diagnostic Scale and the DSM-IV ADHD Rating Scale. Nicotine dependence: Measured by the Fagerström Test for Nicotine Dependence (FTND). Smoking abstinence: self-report confirmed with CO measurement of <8 ppm. Nicotine withdrawal: Withdrawal Scale for Tobacco (WST), Weight	Participants in the OROS-MPH group lost an average of 1.6% of their body weight, while those in the placebo group gained an average of 1.3%. Difference was statistically significant (*p* < 0.001). No significant drug x gender interactions percent weight change. The group receiving OROS-MPH had a lower severity of hunger (*M* = 1.1) compared to the placebo group (*M* = 1.6). Difference was statistically significant (*p* < 0.001).	The study did not do an intention-to-treat analysis along with the completing sample analysis. The use of the nicotine patch may introduce some further bias to the study.
Quilty, 2019 (25)	Canada	Randomized Controlled Trial	To compare the effect of methylphenidate versus cognitive behavioral therapy (CBT) on reducing binge eating episodes in women with BED, as well as the modulating effect of impulsivity	*n* = 49 randomized into CBT group (*n* = 27) and MPH group (*n* = 22). Adult women 19–51 years old. All with BED. BMI larger or equal than 25 kg/m^2^. One third either a mood or an anxiety disorder. None were currently pregnant or breastfeeding, had undergone recent psychotherapy or behavioral treatment for eating/weight, had taken psychotropic medication recently, had severe mental disorders or uncontrolled medical conditions, taking medications affecting weight or contraindicated for methylphenidate.	Patients on the MPH group had weekly appointments for the first 4 weeks, then twice a week for8 weeks. MPH doses were increased from 18 mg/day to 72 mg/day by week 4, and adjusted for side effects, with discharge to a family physician after 12 weeks. Patients on the CBT group had a weekly for 12 weeks lasting 50 min each. Sessions focused on eliminating binge episodes, reducing intake, restructuring cognitions, and preventing relapse.	Binge Eating Behaviors: Frequency of objective binge episodes per week, assessed by a daily binge diary. Quality of Life: QoL inventory Impulsivity: Impulsive Behavior Scale (UPPS-P) BMI	There was a significant decrease in binge episodes in both treatment groups (*F* = 11.9, *p* < 0.001). BMI over time significantly decreased in both treatment groups (*F* = 4.4, *p* < 0.001), but there was a significant difference in BMI between treatment groups at Week 12 with a larger weight loss in the MPH group (*t* = 2.73, *p* = 0.01). There was a significant time × perseverance interaction that modulated objective binge episodes (*F* = 2.10, *p* < 0.02); and a significant time × negative urgency interaction modulating subjective binge episodes (*F* = 1.79, *p* = 0.049).	The sample size is good and supposedly well powered, but subgroup analyses that are non-significant must be analyzed with caution. The sample does not represent males.

All the articles measured at least one appetite/dietetic indicator as an outcome. Three crossover randomized studies evaluated the effect of MPH on food consumption, food cravings, and appetite variables and how this effect interacts with BMI (21), food addiction (23), and binge eating disorder (BED) (22). People with a normal BMI had a significant consumption reduction in snack consumption (*p* = 0.017), appetite ratings (*p* = 0.017), and food cravings (*p* < 0.0001) when receiving MPH compared to placebo. In contrast, in people living with obesity, there was only a snack consumption reduction (*p* < 0.0001), appetite ratings (*p* < 0.007), and food cravings (*p* = 0.008) in women when receiving MPH but not in men (21). Participants with food addiction had higher baseline food cravings and appetite than participants without good addiction (*p* < 0.0001 for both). Regardless of food addiction status, all participants showed a significant decrease in appetite ratings (η^2^ = 0.157, *p* = 0.031) y and food cravings (η^2^ = 0.128, *p* = 0.006) when given MPH compared to placebo. There was only a significant interaction between food addiction and MPH for snack consumption, where participants without food addiction reduced their intake when receiving MPH (η^2^ = 0.276, *p* < 0.0001) (23). In Davis et al. (22), there was a significant decrease in appetite ratings (*p* = 0.002), food cravings (*p* = 0.023), and snack consumption (*p* = 0.002) when participants took MPH, regardless of whether they had BED or not. There was no effect of BED on any of the variables studied. In contrast, Quilty et al. (25) found that the frequency of binging episodes decreased when taking MPH in comparison with CBT (*F* = 11.9, *p* < 0.001) and that this effect had a significant interaction with time (*F* = 2.10 *p* < 0.02).

Other studies replicate similar results. El Amine et al. (18) found that desire to eat (*p* = 0.001), hunger (*p* = 0.001), and prospective food consumption (*p* = 0.003) decreased, and satiety increased (*p* = 0.028) in people with obesity receiving MPH when compared to placebo. Moreover, another study reported a gender x MPH interaction for energy (*F* = 4.9, *p* = 0.01) and carbohydrate (*F* = 8.2, p = 0.02) intake, where males had more considerable reductions than females (24).

In nine out of 10 articles in Category 2 (27–35), weight changes were studied as a side effect. Weight loss is reported in eight articles studying weight loss, while the remaining article reports no changes in weight (27–29, 31–35). In only one article, weight loss was measured and reported in kilograms (35). In this study, the mean weight loss in the MPH group was 0.8 kg versus no weight loss in the placebo group (*p* < 0.05). One study measured the proportion of participants with a weight loss larger than 10% of their baseline body weight (27). The remaining seven articles reported the proportion of participants with any weight loss (28–34). The number of participants who lost weight followed a dose–response pattern. In RCTs, at doses of 54 mg, 0.0–10.1% reported any weight loss, and at doses of 72 mg, the interval of participants losing weight was between 0.0 and 23% (28–32). Adler et al. (27) showed that the number of participants losing over 10% of their initial body weight was 11.1% at any MPH dose (Table 2).

**Table 2:** Effect of MPH on weight, and hunger studied as a side effect.

Article	Country	Design	Objective	Sample characteristics	Intervention	Relevant side effects reported
Adler et al. (27)	USA	Open label Randomized Controlled Trial	To assess the safety of OROS-MPH in the long-term treatment of ADHD in adults.	*n* = 540 Adults between 18–65 years old with ADHD. 48% females	MPH dose was titrated starting at 36 mg/d and escalated up to 108 mg depending on safety. There were two groups: one received the drug for 6 months, and the other for 12 months.	**Weight changes.** Proportion of participants exhibiting more than 10% weight loss increased in a dose–response pattern (1.3% of participants at 36 mg, and 18.1% at 108 mg. 11.1% at any dose). Only 0.9% of the sample gained more than 10% of their initial weight at any dose. This variable did not exhibit a dose–response pattern. **Nausea.** 11.1% of the sample presented with nausea at any dose. This variable did not exhibit a dose–response pattern.
Bron et al. (28)	The Netherlands	Cross-over Randomized Controlled Trial	To evaluate the effect of OROS-MPH in adult executive functions.	*n* = 22 (12 allocated to MPH first and 10 to placebo first). Mean age 30.5 with SD 7.4 years. All adults with ADHD. 22.7% females	For 6 weeks, participants received a titrated MPH dose starting at 36 mg/d for 7 days. 36 mg weekly increments were done until reaching 72 mg for 3 weeks.	A non-quantified weight loss rate of 23% was reported in this study.
Casas et al. (29)	42 European locations (Managed in Germany and Spain)	Randomized Controlled Trial (Phase III)	To determine the efficacy and safety of two doses (54 and 72 mg/d) of OROS-MPH in adults with ADHD.	*n* = 279, (90 in MPH 54 mg, 92 in 72 mg and 97 in placebo)- Adults 18–56 years old with ADHD 45.7–51.1% females	Dose was titrated to 54 or 72 mg according to group starting in 36 mg/d. There was also a placebo group. Dose was increased 7 days after initiation to the required dose. Trial lasted 13-week	**Weight changes.** Dose – response weight-loss was observed (4.1% of participants in placebo group, 10.1% in 54 mg group, and 18.5% in 72 mg group). It was not quantified. **Anorexia**. Dose—response self-reported anorexia was observed (4.1% in placebo, 6.7% in 54 mg group, and 13.0% in the 72 mg group). **Nausea.** Nausea was seen in 8.2% in placebo, 18.0% in the 54 mg group, and 17.4% in the 72 mg group. **Appetite.** Dose – response trend in decreased appetite (5.2% in placebo, 19.1% in the 54 mg group, and 28.3% in the 72 mg group).
Edvinsson and Ekselius (36)	Sweden	Cohort Study	To determine the safety profile of MPH in adults with ADHD over a long period of time.	*n* = 112. 51% of them in treatment. Mean age was 35 years old at the beginning and 42 years old at the end of the study. 46 were taking MPH, 3 were taking MPH and Atomoxetine, and 8 were taking dexamphetamine. 37% females	No actual intervention. Participants with ADHD were followed for 6 years.	**Appetite** In the group taking MPH (n = 46) 28% of the participants reported decreased appetite **Nausea/Vomiting** In the group taking MPH (n = 46), 6.5% reported nausea or vomiting.
Ginsberg et al. (30)	Sweden	Randomized Controlled Trial	To assess the long-term effectiveness and persistence of OROS-MPH related side effects on cognition, motor activity, institutional behavior and quality of life of male adult prison inmates with ADHD.	*n* = 30 (*n* = 15 for placebo and *n* = 15 for MPH group) Adult males between 21 and 61 years old. High prevalence of comorbidity such as substance abuse, antisocial personality disorder, mood and anxiety disorders.	This was a 52-week trial. Dose started at 36 mg for 4 days, then increased to 54 mg for 3 days, and finally to 72 mg for 4 weeks. Those who completed the 4 weeks, entered an open-label extension with a dose of 1.3 mg/kg based on response and tolerability.	No effect on body weight was observed in this study.
Hurt et al. (31)	USA	Randomized Controlled Trial	To explore the effect of OROS-MPH on smoking cessation in adults.	*n* = 80 (40 randomized to each group). Mean age was 38 years in the placebo group and 35.6 years in the OROS-MPH group. 57.8% female	This was a 6-month study comprised by 1 telephone pre-visit, 11 clinical visits and 1 telephone follow-up. Participants were titrated to a dose of 54 mg/d for 2 weeks, and this maximum dose was maintained for 8 weeks with weekly assessments.	**Anorexia** 7.5% of the participants in the MPH group presented anorexia vs. 0.0% of the participants in the placebo group. **Weight changes** 2.5% of the participants in the MPH group lost an unknown amount of weight vs. 0.0% of the participants in the placebo group. **Nausea** 5.0% of participants in the MPH group presented nausea, while only 2.5% of the participants in the placebo group did.
Kis et al. (32)	Germany	Randomized Controlled Trial	To compare the effectiveness and safety of MPH and CBT in adults with ADHD over a 1-year period.	*n* = 419 (randomly assigned to 4 groups: MPH + CBT, MPH + Clinical Management (Clin), Placebo (Pl) + CBT, Pl + Clin). Mean age 35 years old (range of 18–56) Females from 45.3 to 56% depending on group	OROS-MPH dose was titrated to 54 mg/d during a 2-week period and maintained for 8 weeks. Participants attended the clinic weekly for counseling sessions.	**Decreased appetite** Occurred in 22.4% of the MPH group vs. 3.8% of the Pl group (*p* < 0.05) **Nausea** 12.2% of the participants in the MPH group reported nausea vs. 9.6% in the Pl group. Not statistically significant. **Abdominal discomfort** 6.3% of participants in MPH group vs. 2.9% of participants in Pl group. Not statistically significant. **Weight changes** 6.3% of participants in MPH group decreased their weight, while only 1.9% of participants in Pl group. (*p* < 0.05)
Michelsen et al. (35)	The Netherlands	Cohort Study	To assess the cardiovascular side effects of stimulant medications in older adults with ADHD.	*n* = 113 (89 had some pharmacological treatment) age was between 55 and 79 years 57% female	No actual intervention. 44% of the patients had extended release (ER) MPH, 9.7% were taking dexmethylphenidate (DMP), and 7.1% were taking Dexamphetamine (DAM). The observational study lasted 1 year.	**Weight changes** A significant 0.8 kg weight decrease was observed in patients taking MPH (*p* < 0.05). No significant weight changes were observed in other medications.
Retz et al. (34)	Germany	Randomized Controlled Trial	To determine if ER MPH reduces ADHD symptoms and psychopathology in adults with ADHD.	*n* = 162 (84 randomized to MPH ER, and 78 to placebo). Age between 18 and 56 Females 54.8% in MPH ER group, and 43.6% in placebo	MPH ER dose was titrated up to 40–120 mg/d (1 mg/kg maximum) for 2 weeks and then brought up to maximal dose for 6 weeks.	**Weight changes** 48% of participants in MPH group decreased their weight at the maximal dose tolerated, while only 10% of participants in Pl group. **Nausea** 17% of the participants in the MPH group reported nausea vs. 4% in the Pl group.
Retz et al. (33)	Germany	Cohort Study	To describe the safety profile of MPH in adults with ADHD attending a real-world clinic.	*n* = 468 from 126 sites. Age between 18 and 71. Females 42.1%	No actual intervention. Dose was started at 0.23 mg/kg and increased to 0.45 mg/kg as per the clinic protocol.	The study reports weight loss rate of 1.71% and nausea rate of 0.43%.

Regarding other relevant effects, nausea was reported in 7 studies (27, 29, 31–34). Adler et al. (27) reported nausea in 11.1% of the patients at any dose with no dose–response effect. Casas et al. (29) also found no dose–response effect with nausea in 17.4–18.0% of the participants. In cohort studies (33, 34, 36), the rate of nausea was between 0.43–6.5% (Table 2). Three studies reported decreased appetite as a side effect (29, 32, 36). Two were RCTs (29, 32), and one was a cohort study (36). Casas et al. (29) found a dose–response trend in reduced appetite. In this study, the prevalence of decreased appetite was 19.1% at 54 mg MPH and 28.3% at 72 mg MPH. Kis et al. (32) found a prevalence of decreased appetite at 54 mg MPH of 22.4%. The prevalence of decreased appetite in the cohort study was 28% (36). Anorexia was reported in only one article (31). The prevalence of anorexia in this study was 7.5% at a dose of 54 mg (Table 2).

Some of the reviewed studies found slight differences in this response between genders. Women showed more significant reductions in appetite, food cravings, and food consumption in response to MPH than men. This effect is consistent regardless of the presence of BED (22) and food cravings (21, 23). The differential expression of dopamine receptors in distinct brain areas can explain these sex-specific susceptibilities. Women tend to have more D2Rs in the frontal cortex and striatum than men, making them more sensitive to dopamine’s effect on eating behaviors and, therefore, more prone to reduce their food intake due to MPH.

Conversely, males have more dopamine-1 receptors (D1R) in reward-processing areas such as the NAc (37) and probably overeat. Moreover, when depressed, women tend to show more dopamine transporter (DAT) binding, probably making it more susceptible to being inhibited by MPH (37). It is essential to mention that males seem underrepresented in most articles that reach these conclusions. For this reason, more studies in males with well-powered sample sizes are required.

### The mechanism of action of MPH and its effect on eating behaviors and body weight

Research has shown that food intake regulation comprises two mechanisms—a homeostatic hunger-satiety mechanism to regulate energy balance controlled in the hypothalamus and a mechanism that is not driven by energy needs (sometimes called hedonic) that includes hypothalamic control but is mainly regulated in the neocortex and limbic system (38). In addition, a decrease in Dopamine 2 receptors (D2R) expression in the dorsal striatum and NAc has been associated with compulsive food intake in animal models and humans (38, 39).

In addition, the VTA in the midbrain projects neurons to the NAc, forming a complex network that will regulate food’s motivational saliency. Food cues are categorized and prioritized as pleasurable and compelling in these brain areas. According to Nicola (38), food’s rewarding effect can be classified into three different components: the motivational component (wanting), the hedonic component (liking), and the learning component (reinforcement). The motivational component of eating has been related to the dopaminergic pathways, while there is evidence that the hedonic component has an opioergic regulation (38, 40).

The brain’s dopaminergic systems and conditioned learning drive food-seeking behaviors in humans. This means that even without hunger, different stimuli (i.e., smells, memories, or the sight of food) can motivate an individual to look for food, even when it implies a significant effort. In addition, dopaminergic neurons in these circuits appear to be regulated by hormones that regulate energy balance (homeostatic mechanisms). Neuropeptide Y (NPY), ghrelin, orexins, and agouti-related peptide (AgRP) have been seen to increase dopamine release, while glucagon-like peptide 1 (GLP-1), insulin, and leptin decrease it (38, 40).

In rodents, Sucrose has been shown to stimulate dopamine transmission in the ventral striatum and olfactory bulb—cues paired with sucrose stimuli condition dopamine release in these brain regions. The effects of sucrose in the dopaminergic pathways have been compared to the effects of several drugs on the same areas. The effects appear to differ in the higher speed at which dopamine activity subsides after sucrose is used (39).

**Figure 5:**
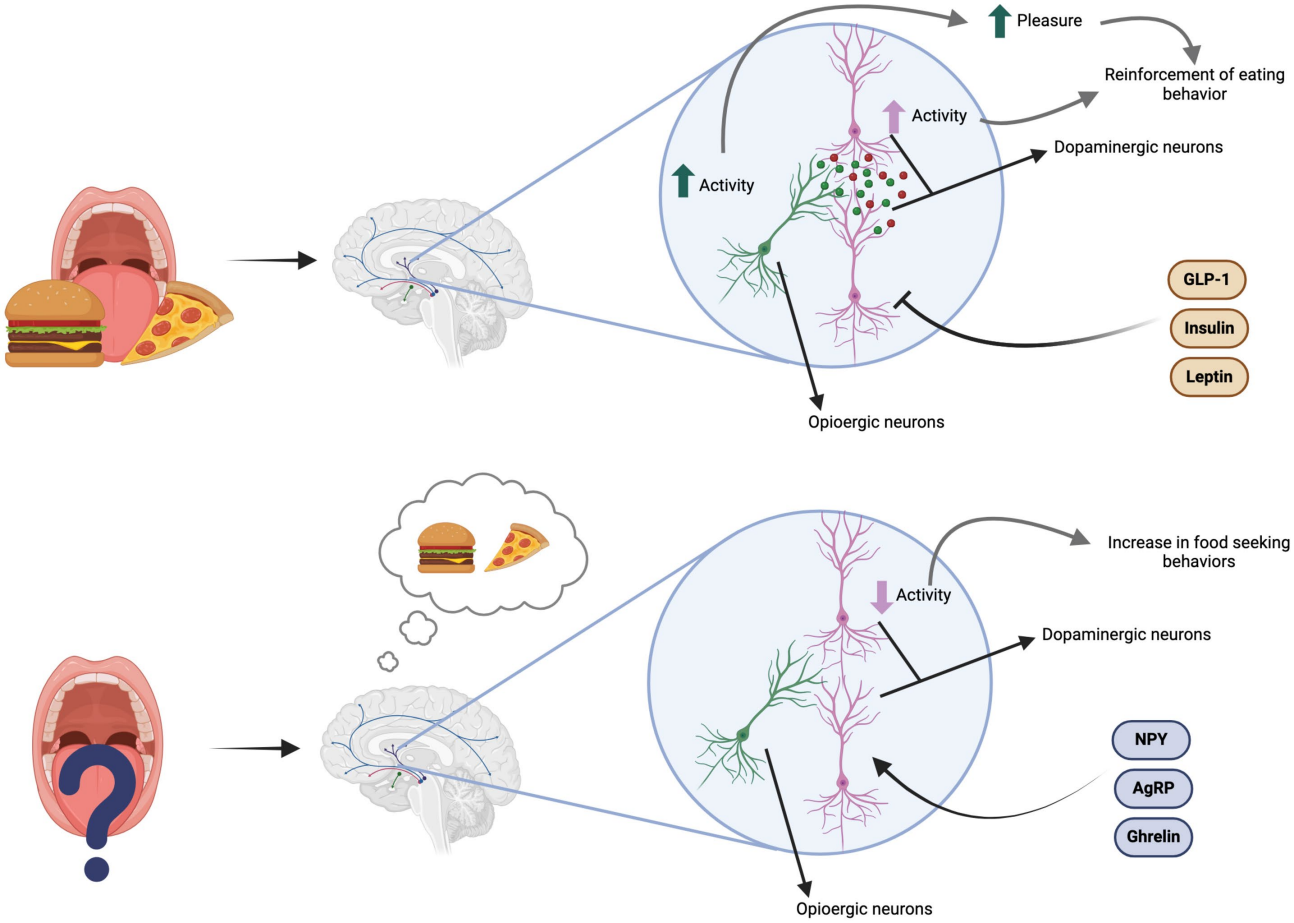
The dopaminergic model of appetite regulation explains how the brain responds to food. After consuming a palatable meal, opioid and dopaminergic activity in the mesolimbic pathway increase, enhancing pleasure and reinforcing eating behavior. Anorexigenic peptides inhibit this dopamine activity. In the absence of palatable food, dopamine levels remain low, but the sole thought of pleasurable food can trigger food-seeking behaviors to restore dopamine levels. Orexigenic peptides stimulate dopamine activity (38–40).

Pleasurable stimuli activate the opioid system. Consuming palatable and calorie-dense foods stimulates *μ*-opioid receptors in the NAc. Activating the opioid system increases the motivational salience of food through a Pavlovian conditioning mechanism. Cues that remind the individual of a pleasurable eating experience can further reinforce dopamine release (38, 40). Figure 5 depicts the mechanisms mentioned above.

Disrupted dopaminergic signaling, including decreased D2R expression in areas of the reward network such as the dorsal striatum, the VTA, and the NAc, translates into reduced activity in the orbitofrontal cortex and the cingulate gyrus. Since these systems regulate compulsive eating (39), their dysregulation can lead to overeating highly palatable foods (39, 40). Given that MPH inhibits dopamine reuptake, it follows that enhancing dopamine’s action in these areas could modulate compulsive eating behaviors. Notably, MPH has been shown to decrease the intake of dietary fats and carbohydrates, suggesting a shift in macronutrient preference toward lower-fat options (24). This effect could help people struggling to lose weight to improve their food choices and modify their food composition. While this review focuses on the effects of MPH in adults, literature has also found similar effects on teenagers (41).

As previously mentioned, MPH inhibits dopamine and norepinephrine synaptic reuptake and is available in various pharmaceutical presentations (Figure 1). The literature shows that MPH can reduce food intake and weight. This effect is seen in articles that aim to determine if MPH can help adult patients lose weight and reduce their intake (Category 1) and in articles that evaluate different research questions regarding the use of MPH in adults (Category 2). Further exploring its potential effects on weight, body composition, and food intake could help increase the availability of safe and tolerable pharmacological interventions to treat obesity or excess weight.

MPH’s effect of increasing dopaminergic activity in the ML, MC, and MS pathways can suppress appetite and reduce food intake. Increased dopamine release in these brain areas implies that the motivational salience of food will be reduced (39, 42). As a result, people with obesity or overweight taking MPH could reduce their energy intake and improve their food choices (18, 41).

MPH also appears to reduce appetite and food intake by modulating olfactory sensitivity (18). These findings are interesting because the literature on obesity and olfaction has shown that individuals with obesity seem to discriminate smells less than their normal-weight counterparts. Impaired olfaction may delay satiety cues, and olfactory cues could influence food choices. It is essential to mention that it is impossible to establish a causal relationship between olfaction and obesity because there may be a bidirectional association – impaired olfaction may alter intake and metabolism. Still, obesity may, in turn, affect how the brain perceives smells and detection thresholds (43, 44).

Olfactory cues seem tightly linked to dopaminergic processing in different brain regions. Sorokowska et al. (45) have shown that food odors can increase dopaminergic activity in reward circuits such as the anterior cingulate cortex, the putamen, and the insula, thus influencing eating behaviors. These results seem to be supported by Rampin et al. (46), who show that food odors can further increase dopaminergic transmission in the ventral striatum.

Interestingly, the results on olfactory sensitivity in participants with ADHD seem to be discrepant. Some studies have replicated olfactory impairment in children with ADHD (47). However, another study even showed that MPH cessation in children with ADHD improves olfactory discrimination (48). More work in this area is needed to determine the role of olfaction in developing unwanted eating behaviors. As it is, MPH’s dopamine reuptake inhibition could reinforce increased olfactory detection and thus improve eating behaviors. Also, while MPH seems to have a dose–response effect on appetite, all doses used in the reviewed studies decreased appetite. This means that moderate and high doses of MPH reduced energy intake, with a notable reduction in the consumption of highly palatable foods. This effect is replicated in older literature (49).

### Clinical considerations and safety issues

While promising as a potential weight-loss intervention, it is important to mention that MPH has been associated with increased cardiovascular risk in patients who are susceptible to heart conditions (50). Moreover, some studies in children with ADHD have shown that MPH has proarrhythmic properties (51). A prospective cohort study with a three-month follow-up in 100 Iranian children with ADHD between 6 and 11 years old found that children taking MPH had significantly higher systolic and diastolic blood pressures and increased heart rates. There were no significant differences in the cardiac output, QT interval, and left ventricular mass. Clinically irrelevant changes in systolic and diastolic functions were also seen in children taking MPH, but the drug was determined to be safe (52).

A retrospective study on 26,710 individuals between 12 to 60 years without ADHD using MPH matched to 225,672 controls found that there was a 41% increased risk of cardiovascular events in the group using MPH (50). Another retrospective study on 43,999 new MPH users matched to 175,955 non-users found an 84% increased risk for sudden death or ventricular arrhythmia and a 74% risk of all-cause mortality in MPH users. There was no significant risk of stroke or myocardial infarction, and there was no significant dose–response effect or extended vs. immediate release effect (53).

In addition, a systematic review and meta-analysis analyzing the cardiovascular risk associated with medications used in ADHD gathered 19 observational studies and nearly 4 million participants from different age groups. The risk of cardiovascular events was not significant in stimulant users, non-stimulant users, or users of any age group, suggesting that the risk of cardiovascular events in stimulant users is the same as the risk in the overall population (54).

The literature shows mixed results regarding the cardiovascular risks linked to MPH. Since people with obesity have a higher rate of heart comorbidities than their normal-weight counterparts, further studying the safety profile of MPH in people with obesity and overweight is of prime importance before considering it a therapeutic option in this population. It is also important to consider gender and ethnic differences in dopamine receptor expression to fully understand the plausibility of using MPH as a treatment for obesity and overweight.

## Discussion

Since the early 2000s, several studies have found that MPH can lead to weight loss in individuals. A meta-analysis in 2007 of 8 randomized controlled trials found that methylphenidate treatment resulted in an average weight loss of 2.03 kg compared to placebo (55). These effects appear to be mediated by reduced appetite and food intake, a competitive regulation of dopamine without the action of eating (49, 55). This review has found similar effects in newer studies. The selected studies indicate that the use of MPH can produce a modest weight loss and appetite suppression, particularly through its effects on the brain’s hedonic and sensory processing pathways and that this effect appears more pronounced in women. Side effects, such as nausea and anorexia, may also contribute to these outcomes.

The interpretation of these findings is limited by several factors: study heterogeneity, small sample sizes, and lack of long-term data make it challenging to generalize results. Additionally, none of the reviewed studies evaluated MPH in combination with lifestyle or dietary interventions, which are commonly prescribed together with weight-loss drugs in clinical practice. MPH’s association with cardiovascular risks highlights the need for caution, especially in patients with obesity who may already have an elevated risk of heart disease. While MPH shows potential as an adjunct therapy for weight management, further research is essential to confirm its safety and efficacy in broader, more diverse populations and to determine its suitability for long-term use.

Some examples of real-world include one using a Phentermine + Topiramate combination for the treatment of obesity in adolescents included a lifestyle intervention for both placebo and experimental groups. This study showed a maximum BMI loss of 10.44% after 56 weeks of treatment (56). Another trial using glucagon-like peptide-1 (GLP-1) agonists in patients with type-2 diabetes in the “real world” found that over 67% of the participants lost more than 5% of their initial body weight at 72 weeks without explicitly offering lifestyle interventions, and mean weight loss was 2.2% (57). This is comparable with the magnitude of weight loss found in the articles in this review, which was around 1.6% (26). Also, the proportion of participants losing over 10% of their initial body weight was around 11% in Adler et al. (27). However, another article using GLP-1 agonists plus lifestyle interventions found that an exercise intervention increased the number of participants losing weight 3.7 times compared to the control group and that exercise protected participants from regaining weight after treatment (58).

Another area that limits discussion is that it is difficult to compare the selected studies given their heterogeneity and that three articles appear to come from the same cohort (21–23). Furthermore, measurements, doses, and MPH presentations are not standardized across the studies. Also, it is essential to remember that none of the studies in Category 1 addressed any adverse effects of MPH that may become relevant in people with obesity.

MPH is not the first drug with noradrenergic/dopaminergic activity to be considered to promote weight loss in individuals with obesity or overweight. Amphetamine derivatives, phentermine, bupropion (all enhancing norepinephrine and dopamine activity through different mechanisms), and sibutramine (a serotonin and norepinephrine reuptake inhibitor), among others, have been used alone or in combination to promote weight loss. Similar drugs that are currently approved for weight loss come in combination. Examples include Phentermine + Topiramate (an antiseizure drug with multiple targets) and Bupropion + Naltrexone (a *μ*-opioid receptor antagonist used in higher doses to treat alcohol cravings) (20). Given its similar pharmacodynamic profile and moderate weight-loss-inducing properties, MPH could be a good candidate for further study. While MPH does enhance dopamine activity in reward-processing brain areas and the evidence does show that MPH can decrease weight and promote anorexia, more studies are needed to fully uncover adverse effects in people with obesity who may be at risk of cardiovascular events, the optimal doses to promote weight loss in different populations, and its potential to be combined with other drugs.

## Conclusion

Methylphenidate appears to suppress appetite and reduce food intake in adults with obesity or overweight. This effect appeared to be more pronounced in women. Given the current state of the evidence, it is not possible to determine if men are less sensitive to the anorexigenic effects of MPH or if the sample was underpowered. MPH also seems to influence macronutrient preferences, reducing fat and carbohydrate intake. These effects could be mediated by increased dopamine levels, which affect the reward value of food. Overall, MPH shows promise as a potential pharmacological intervention for weight management in obese and overweight individuals.

Current studies are limited by small sample sizes, design heterogeneity, short follow-up periods, and lack of integral accompanying interventions. To build a robust evidence base, future research should prioritize large-scale randomized controlled trials focusing on the long-term efficacy and safety of MPH in diverse populations. Studies assessing cardiovascular risks in individuals with obesity and MPH’s impact over extended periods are especially important. Furthermore, analyzing the effect of MPH in combination with lifestyle modifications or other anorexigenic/weight-loss medications could provide further answers into its possible role within a comprehensive weight management strategy. Understanding optimal dosing and the role of gender differences in MPH’s effects on appetite and weight regulation also remain unanswered issues that need future addressing.

## Data Availability

The original contributions presented in the study are included in the article/supplementary material, further inquiries can be directed to the corresponding author.
